# Phylum‐level diversity of the microbiome of the extremophilic basidiomycete fungus *Pisolithus arhizus* (Scop.) Rauschert: An island of biodiversity in a thermal soil desert

**DOI:** 10.1002/mbo3.1062

**Published:** 2020-06-01

**Authors:** Ken Cullings, Matthew B. Stott, Nicole Marinkovich, Julia DeSimone, Shilpa Bhardwaj

**Affiliations:** ^1^ JQ Division NASA‐Ames Research Center Moffett Field California USA; ^2^ School of Biological Sciences University of Canterbury Christchurch New Zealand

**Keywords:** 16S ribosomal RNA gene, geothermal, microbiome, *Pisolithus*, Yellowstone National Park

## Abstract

We used high‐throughput DNA sequencing methods combined with bio‐geochemical profiles to characterize the internal environment and community structure of the microbiome of the basidiomycete fungus *Pisolithus arhizus* (Scop.) Rauschert from soils within a geothermal feature of Yellowstone National Park. *Pisolithus arhizus* is unique in that it forms closed fruiting bodies that sequester visible sulfur within. Fourier transform infrared spectroscopy (FTIR) analysis demonstrates that the *P. arhizus* fruiting body also concentrates copper, manganese, nickel, and zinc and contains pure granular silica. Gas chromatography‐mass spectrometry (GC‐MS) analysis indicates an environment rich in hydrocarbons. Oxygen probe analysis reveals that zones of up to 4× atmospheric oxygen exist within nanometers of zones of near anoxia. Analysis of microbial community structure using high‐throughput DNA sequencing methods shows that the fruiting body supports a microbiome that reflects the physiochemical environment of the fruiting body. Diversity and richness measures indicate a microbiome that is significantly richer and more diverse than that of the soils in which *P. arhizus* grows. Further, *P. arhizus* sporocarps are enriched significantly in Proteobacteria (primarily *Burkholderia*) Gemmatimonadetes, Bacteroidetes, Verrucomicrobia, Nitrospirae, Elusimicrobia, and Latescibacteria (WS3) while soils are enriched in Actinobacteria (primarily *Mycobacterium*), Dormibacteraeota (AD3), and Eremiobacteraeota (WPS‐2). Finally, pairwise % similarity comparisons indicate that *P. arhizus* harbors two lineages that may represent new groups in the candidate phylum radiation (CPR). Together, these results demonstrate that *P. arhizus* provides a novel environment for microbiome studies and provides for interesting hypotheses regarding the evolution, origins, and functions of symbioses and novel microbes.

## Graphical abstract

This work describes the microbiome of the thermophilic fungus *Pisolithus arhizus*. *Pisolithus* *arhizus* creates an energy‐rich island refugium habitat in a thermal soil desert that harbors microbial community that is distinct from parent soils and includes deeply diverging novel lineages. The results demonstrate that *P. arhizus* provides for interesting hypotheses regarding the evolution, origins, and functions of symbioses and novel microbes.

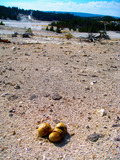

## 1 INTRODUCTION

Fungi have contained microbial symbionts since their inception over a billion years ago (Bengtson et al., 2014, 2017). Though the microbiomes of extant vesicular‐arbuscular mycorrhizal (AM) associations of the Glomeromycota have been the subject of considerable study (e.g., Naito, Morton, & Pawlowska, 2015; Salvioli et al., 2016; Torres‐Cortes, Ghignone, Bonfante, & Schussler, 2015) only recently has attention turned to the microbiomes of mushroom‐forming fungi, basidiomycetes. Mushrooms (fruiting bodies) can play host to an array of microorganisms, including Proteobacteria, Bacteroidetes, and Actinobacteria (Barbieri et al., 2005; Benucci & Bonito, 2016; Pent et al., 2018). Their functionality in this endosymbiotic setting is only now coming to light via genomics sequencing methods. Data indicate that microbes likely enhance mycelial growth and fruiting body formation, nitrogen nutrition, and spore dispersal, influence gene expression, and act as biocontrol agents through mycotoxin production (e.g., Bahram, Vanderpool, Pent, Hiltunen, & Ryberg, 2018; Barbieri et al., 2010; Cho, Kim, Crowley, & Cho, 2003; Deveau et al., 2018; Lackner, Partida‐Martinez, & Hertweck, 2009; Torres‐Cortes et al., 2015).

It has been hypothesized that host genetics, fruiting body type, and mode of nutrition could all influence microbiome community structure in fungi (Barros et al., 2007; Barros, Venturini, Baptista, Estevinho, & Ferreira, 2008; Pent et al., 2018; Rinta‐Kanto, Pehkonen, Sinkko, Tamminen, & Timonen, 2018). Evidence for the latter relationships comes in good part from comparisons of saprophytic and ectomycorrhizal fungi which differ in their mode of nutrition; saprophytic fungi rely on carbon obtained from the breakdown of exogenous substrates, while ectomycorrhizae are close symbiotic relationships between plants and fungi in which the fungal partner provides all fixed nitrogen required by the phytobiont in return for up to 90% of the carbon fixed by the phytobiont via photosynthesis (Smith & Read, 2010). Data from these comparisons indicate that the fruiting bodies of saprophytic and ectomycorrhizal basidiomycetes can be differentially dominated by populations of representatives of Archaea, Actinobacteria, Gammaproteobacteria, Bacilli, and Clostridia (Barbieri et al., 2005; Benucci & Bonito, 2016; Pent et al., 2018; Quandt et al., 2015; Rinta‐Kanto et al., 2018; Zagriadskaia, Lysak, Sidorova, Aleksandrova, & Voronina, 2013).

**FIGURE 1:**
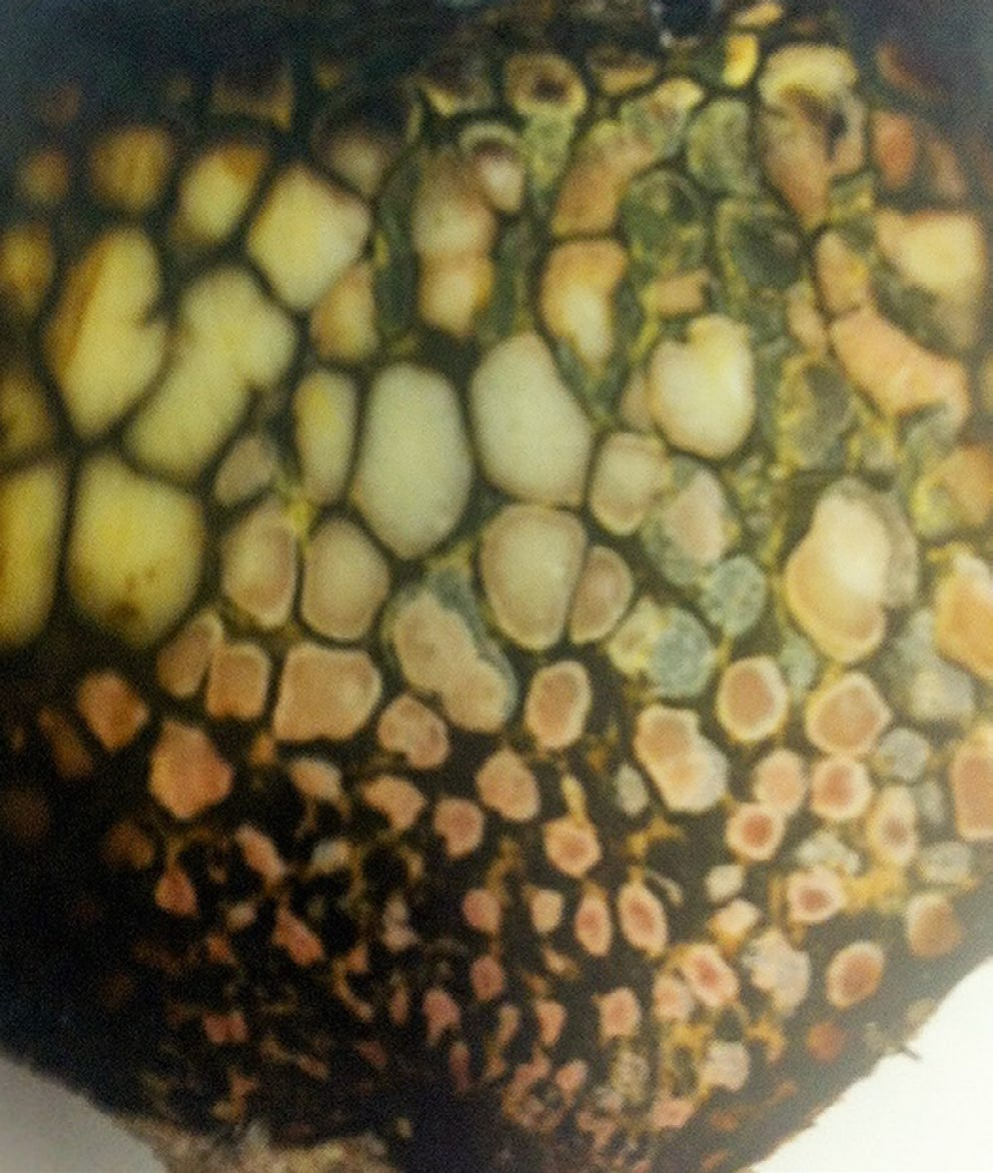
Cross‐section of a *Pisolithus arhizus* individual depicting granular inclusions set amidst hydrocarbon slurry and spore mass. Colors represent microbes (pink) and algal (green) components of the microbiome that inhabit the island refugium habitat created by conditions within fruiting individuals. FTIR analysis indicates that inclusions are sulfur‐coated silica granules

In this study, we investigated the microbiome of the basidiomycete fungus *Pisolithus arhizus* (Boletales). *Pisolithus arhizus*, basonym *Pisolithus tinctorius* (Lebel, Pennycook, & Barett, 2018), is a terrestrial fungal extremophile that can grow in soils associated with acid‐thermal hot springs and acid mine tailings at pH and temperature extremes that are beyond the threshold of survivability for most other organisms (Cullings & Makhija, 2001; Marx & Artman, 1979; Walker, 1989). *Pisolithus arhizus* is peculiar among basidiomycete fungi in that the fruiting bodies of this fungus are closed with a hard outer shell (peridium) and accumulate elemental sulfur into the gleba, the spore‐producing “tissues” (Figure 1) (Muncie, Rothwell, & Kessel, 1975). Mushroom growth is hydraulic; hence, the fruiting bodies contain a considerable amount of water through much of their development and likely also contain CO_2_ from fungal cellular respiration. We have hypothesized that because of these processes and the presence of the peridium acting as a barrier to gas diffusion, there could be anaerobic regions within the fruiting body. If present, these would help to create gas and chemical gradients and transitions, and also both oxidized and reduced forms of sulfur and CO_2_ which could be utilized for microbial metabolism and growth (Cavanaugh, McKiness, Newton, & Stewart, 2006). Before this study, this potential had not been explored in any basidiomycete fruiting body, open or closed. Further, while *P. arhizus* is often ectomycorrhizal, our work previous with *P. arhizus* in Yellowstone National Park (YNP) demonstrates that this fungus does not form mycorrhizal symbioses in these acidic thermal soils even when potential plant hosts are living within just a few meters of *P. arhizus* fruiting bodies (Cullings & Makhija, 2001). Therefore, an alternative carbon source is required. This observation has prompted us to investigate whether *P. arhizus* incorporates a microbiome that could provide for alternative energy sources.

In this study, we used gas chromatography‐mass spectrometry (GC‐MS), FTIR, and ICP‐MS to characterize the chemical and elemental environment within *P. arhizus* fruiting bodies collected from acidic thermal soils in YNP. Also, we used in situ O_2_ probe analysis to test the hypothesis that O_2_ depleted zones would exist within *P. arhizus* fruiting bodies. Finally, we used high‐throughput DNA sequencing of the 16S ribosomal RNA gene (itags) to test the hypotheses (a) that the microbiome of *P. arhizus* would be unique to that of parent soils, (b) that it would be comprised of chemosynthetic bacteria that could provide energy to the mycobiont and (c) that the *P. arhizus* microbiome may harbor novel microbes, novel perhaps to the phylum level.

## 2 MATERIALS AND METHODS

### 2.1 Sample collection

This study was conducted at the Norris Annex, adjacent to Norris Geyser Basin in Yellowstone Park at the following GPS coordinates: Site 1: Lat: 44.711488 Long: −110.552502; Site 2: Lat: 44.731004, Long: −110.699787; and Site 3: Lat: 44.740515 Long: −110.699830. These are the same sites used in our previous *P. arhizus* study (Cullings & Makhija, 2001), and the full site characteristics are described therein. All soils and *Pisolithus* sporocarps were collected aseptically in sterile 50 ml centrifuge tubes and plastic bags, respectively.

### 2.2 Bio‐geochemical characterization of soils and *Pisolithus* sporocarps

#### 2.2.1 O_2_ measures

A Unisense oxygen microsensor (OX‐500, 500‐micrometer tip) connected to a Unisense Microsensor Multimeter and micromanipulator mounted on an in situ stand was used to measure oxygen gradients within *P. arhizus* sporocarps. Data were analyzed using the SensorTrace Suite Profiling Module. Three measurements were taken within each of three *P. arhizus* individuals, in situ. The sensor was allowed to equilibrate for 2 hr before calibration. On‐site calibration was performed using the CAL300 calibration chamber for atmospheric partial pressure, and an anoxic solution of 0.1 M sodium ascorbate, 0.1 M sodium hydroxide for a zero reading as per the manufacturer's instructions. The sensor was connected to the microsensor in situ stand (model IS19) with a micromanipulator (model MM‐33) allowing easy control of probe movement. Each *P. arhizus* sample was harvested and placed immediately on its side, on a platform beneath the sensor probe, with the tip of the probe within 5 mm of the surface of the sample. The peridium was punctured with a sterile needle, and the probe was immediately inserted into the opening. Readings were taken every 0.02 s as the probe progressed through the sample slowly and steadily for a total of 3,500–4,700 readings along 340–345 mm transects replicate through each of the three *P. arhizus* individuals. The data collected in this study were analyzed by importing into an Excel spreadsheet, where each set of readings was charted on an *x*–*y* scatter plot. High and low points were noted for each data set, and standard error calculated for high and low points within each *P. arhizus*, and among all nine data sets. The results are presented graphically.

#### 2.2.2 Elemental analysis

We performed quantitative ICP‐MS analysis on nine *P. arhizus* individuals. We compared the elemental composition of the *P. arhizus* sporocarps to that of parent soils. The analysis was performed by GNS Science, in Taupo, New Zealand. We compared three groups of three *P. arhizus* individuals to three samples from three corresponding soil plots for a comparison of nine *P. arhizus* to nine soil samples. *Pisolithus arhizus* tissue (approximately 1 g) was excised and weighed into a Teflon beaker, with all weights recorded to three decimal points. To the beaker, 4.0 ml of 1:1 HNO_3_ (trace‐metal grade A509; Fisher Scientific) and 10.0 ml of 1:4 HCl (trace grade H1196; Fisher Scientific) were added and beakers were covered with a watch glass. The *P. arhizus* tissues and soils were then digested at 90–95°C. Samples were then refluxed for 30 min and then allowed to cool at room temperature. The digested samples were cooled then transferred to a 100 ml volumetric flask and diluted to 100 ml with de‐ionized H_2_O for elemental analysis. Then, 20 ml of this diluted sample was transferred to a 50 ml volumetric flask and the sample was further diluted to 50 ml with de‐ionized H_2_O. The chemical/metallic composition of the samples was then determined by inductively coupled plasma (ICP) mass spectrometry (Thermo Scientific™ iCAP™ 7600 ICP‐OES). Data were analyzed using Student's *t* test performed manually.

Fourier transform infrared spectroscopy (FTIR) was performed to determine the composition of the granular inclusions within three *P. arhizus* individuals stored in a −80°C freezer and on three samples prepared for analysis while fresh in the field. Frozen samples were dehydrated in an Economy Oven model 25EG (Precision Scientific) at 46°C until samples were at a constant weight. Samples that were sectioned prior to dehydration were dehydrated overnight, whole *P. arhizus* were dried for five days in drying oven. Fresh samples were dried in a NutriChef PKFD12 electric countertop food dehydrator overnight in the field. Dried samples were sectioned based on visible differences and ground in a mortar and pestle into a fine powder. The fine powder was run on two different FTIR instruments: Spectrum Two (PerkinElmer) equipped with a diamond ATR sampling module (PerkinElmer) and ALPHA FTIR Spectrometer system (Bruker) equipped with a diamond ATR sampling module (Bruker). The system was equipped with version 7.5 OPUS software (Bruker). The library search was performed using the version 15.0.145.0 KnowItAll software (Bio‐Rad Laboratories, Inc.) against the full FTIR library which comes with the KnowItAll software.

#### 2.2.3 Gas chromatography‐mass spectrometry

We performed a nonquantitative analysis of the biochemistry inside nine *P. arhizus* individuals. Frozen samples were surface sterilized then ground by mortar and pestle with liquid nitrogen. 152 ± 5 mg of sample was weighed out into 10 ml headspace vial and kept at −80°C until volatile analysis. The rest of sample was lyophilized and then blended in a bullet blender bead homogenizer for 5 min. Biphasic extraction was applied to the samples as follows. Cold MTBE (methyl tert‐butyl ether) mix (MTBE: Methanol:Water, 6:3:1) was added in a ratio of 1.2 ml of solvent mix per mg of sample, and vortexed for 1 hr at 4°C, followed by addition of water of one‐fourth of the volume of MTBE mix and another 15 min vortexing. Then the mixture was subjected to centrifugation at 3,000 × *g* for 20 min. The lower polar phase was collected for polar compound analysis and stored at −80°C until analysis.

The samples in the headspace vial were incubated for 5 min at 70°C. Volatile compounds were extracted by a 65 μm Polydimethylsiloxane/Divinylbenzene SPME fiber (Sigma‐Aldrich) at 70°C for 40 min with agitation, and then desorbed at 250°C for 5 min into a DB‐WAXUI column (30 m × 0.25 mm × 0.25 μm, Agilent) in a Trace1310 GC (Thermo) coupled to a Thermo ISQ‐LT MS. The inlet was set to splitless mode during desorption. The oven temperature program started at 40°C held for 3 min, increased to 240°C at 8°C/min, and held for an additional 10 min at 240°C. Detection was completed under electron impact mode, with a scan range of 35–400 atomic mass units (amu) and a scan rate of 5 scans/s. The transfer line and source temperatures were 250°C.

Polar extract of biphasic extraction (200 μl) was dried under nitrogen, resuspended in 50 μl of pyridine containing 25 mg/ml of methoxyamine hydrochloride, incubated at 60°C for 45 min, sonicated for 10 min, and incubated for an additional 45 min at 60°C. Next, 50 μl of N‐methyl‐N‐trimethylsilyltrifluoroacetamide with 1% trimethylchlorosilane (MSTFA + 1% TMCS, Thermo Scientific) was added and samples were incubated at 60°C for 30 min, centrifuged at 3,000 × *g* for 5 min, cooled to room temperature, and 80 μl of the supernatant was transferred to a 150 μl glass insert in a GC‐MS autosampler vial. Metabolites were detected using a Trace 1310 GC coupled to a Thermo ISQ‐LT MS. Samples were injected at a 10:1 split ratio. The inlet was held at 285°C, and transfer line and ion source were held at 300 and 260°C. The separation was achieved on a 30 m TG‐5MS column (Thermo Scientific, 0.25 mm i.d., 0.25 μm film thickness) with a 1.2 ml/min helium gas flow rate, and the program consisted of 80°C for 30 s, a ramp of 15°C per min to 330°C, and an 8 min hold. Masses between 50–650 amu were scanned at 5 scans/s after electron impact ionization. QC samples that were pooled from the whole sample set were injected after every six samples.

### 2.3 Molecular methods

DNA was isolated using the MoBio Power Soil DNA isolation kit according to the manufacturer's instructions. Nine DNA isolations were performed on each of nine *P. arhizus* individuals. For DNA extraction from soils, we sampled three samples from the area immediately surrounding nine *P. arhizus* individuals for a total of 27 soil samples. Data are reported on a per fruiting body and per soil plot basis. To maximize the likelihood that the fruiting bodies sampled were indeed individual “islands” soils surrounding fruiting bodies were screened for the presence of *P. arhizus* using ITS‐RFLP and fruiting bodies were collected for analysis only when areas between fruiting bodies lacking this fungus were detected. Also, we amplified the nuclear Internal Transcribed Spacer region (ITS‐2) of each *P. arhizus* individual. We used the ITS3/ITS4 primers in the PCR and sequencing reactions and utilized PCR conditions previously described (White, Bruns, Lee, & Taylor, 1990). This was undertaken to ensure that we were analyzing a single species of *Pisolithus* in our study.

The microbiome community compositions of the soil and *P. arhizus* were determined via 16S rRNA gene sequence amplicon sequencing (itags). For this analysis, PCR reactions were undertaken using sporocarp and soil extractions using the PHusion High‐Fidelity PCR Master Mix (New England Biolabs). Libraries were generated using the TruSeq DNA PCR‐Free Prep Kit (Illumina) and quality was assessed using Qubit 2.0 on a Thermo Scientific Fluorometer and Agilent Bioanalyzer 2100 system. The library was sequenced using an IlluminHiSeq2500 platform. 16S itags for the V3/V4 regions were generated by Novogene Corporation. Sequences were assembled using FLASH V1.2.7 (Grayston & Wainwright, 1988), and data were quality filtered using QIIME V2 using the default parameters (Bolyen et al., 2019). Chimeras were removed using UCHIME (Edgar, Haas, Clemente, Quince, & Knight, 2011). Sequences were analyzed using UPARSE v7.0.1001 (Edgar, 2013) and sequences with >97% similarity were clustered as OTUs. Multiple sequence alignments were performed using MUSCLE V3.8.31 (Edgar, 2004). Taxonomic annotation was accomplished using the GreenGene Database version 13_8 (included in the QIIME software package mentioned above), based on the RDP Classifier v2.2, and also by using QIIME‐compatible SILVA (Yilmaz et al., 2014) and BLAST (Altschul et al., 1997). Alpha and Beta diversity measures described below were performed also using QIIME.

Where the above analyses of microbial taxa in *P. arhizus* samples were found to contain OTUs that defied classification or contained entities of special interest using GreenGene and BLAST, we used a combination of PCR followed by cloning/Sanger sequencing of partial 16S rRNA gene PCR products to further investigate the sequence similarity. PCR was performed using bacterial primers 27f/1492r (DeLong, 1992) which produces near full length 16S rRNA gene sequences and contains hypervariable regions V2–V9. PCR reactions were carried out using 24 PCR cycles with an annealing temperature of 60°C, and elongation of 1 min with a final elongation stop of 5 min. PCR products were cloned using the Qiagen PCR Cloning plus Kit, plated with kanamycin selection and blue/ white screening, and modified by the protocol by adding 5 µl of sample to the ligation (Cullings & Hanely, 2010). Clones were sequenced by Sequetech Corp. Sequences were aligned using ClustalW and manually checked. Sequences were assessed for chimeras using DECIPHER (Wright, Yilmaz, & Noguera, 2012). Only portions of the amplified 16S gene that contained sequences that were produced in both directions, overlapped and confirmed were used in subsequent analyses. This resulted in sequences 850–900 bp in length and containing hypervariable regions V4–V9.

Determination of possible unique identity to the phylum level was accomplished by pairwise % similarity comparisons to sequences in GenBank (Sayers et al., 2020) using established % similarity thresholds (Yarza et al., 2010). To rule out external contamination, we took swipes from all lab surfaces and from inside the laminar flow hood and clean box used for cloning and PCR and subjected these swipes to PCR amplification of 16S rRNA genes. Further, we ran reaction blanks on all individual reagents in the analysis stream from DNA prep to cloning. All reagents were negative, and no swipes from any surface produced sequences that matched any of the 16S rRNA gene sequences obtained from *P. arhizus* individuals. At least two separate and independent DNA preps, amplification, cloning, and sequencing analysis streams of *P. arhizus* fruiting bodies were performed by different people. Only sequences confirmed by these independent streams were used in our subsequent analyses.

The communities were analyzed using standard ecological measures. Good's Coverage was used to estimate the percent of total species represented in the sampling. ANOSIM (a nonparametric analysis of similarity) and analysis of molecular variance (AMOVA) were used to detect differences in populations based on molecular markers. Bray Adonis was used to further assess variance between populations. Principal component analysis was used as a method of orthological transformation to convert possibly correlated data points into principal components which can be visualized and samples grouped graphically. Taxon richness was assessed using Rarefaction measures of observed OTUs. Shannon and Simpson's Diversity indices were used as diversity measures that account for species abundance, numbers, evenness, and richness. The Mann–Whitney *U* test (a nonparametric version of the *T* test) was used to determine whether the two communities were derived from the same population. The above analyses were undertaken using QIIME as indicated previously in this section.

Finally, we conservatively inferred the metabolic function of dominant OTUs and sequences obtained via the cloning methods and controls described above by comparing our generated 16S rRNA gene sequences to closest GenBank matches of cultivated strains via BLAST. We only undertook these predictions where OTUs had >95% sequence similarity (and 100% coverage) with previously isolated and characterized microbial taxa.

## 3 RESULTS

### 3.1 Bio‐geochemical analyses of soils and *Pisolithus* sporocarps

**FIGURE 2:**
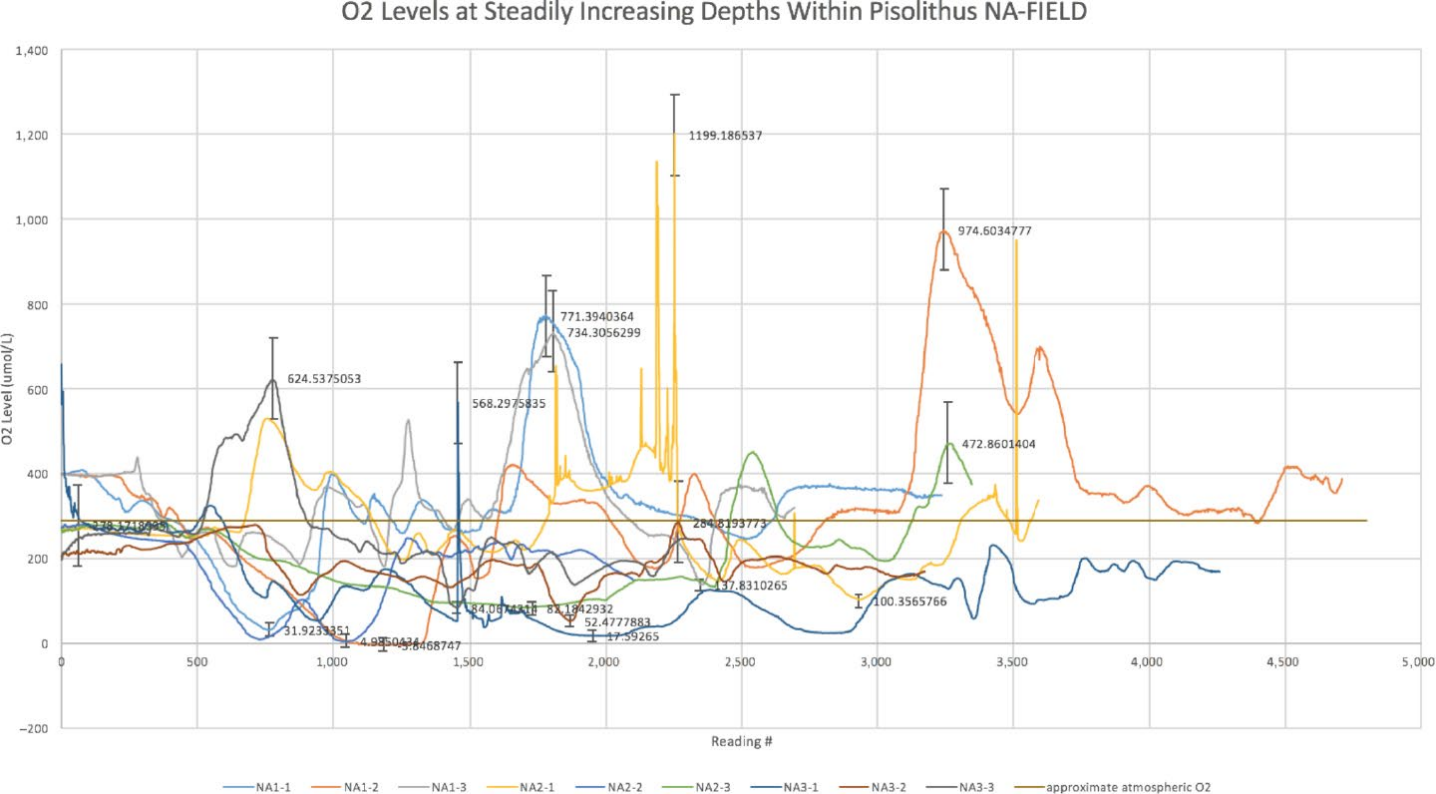
Oxygen measures performed through individual *Pisolithus arhizus* fruiting bodies, in situ. Measures of all individuals were performed on the same day. The *x*‐axis represents a position just inside the peridium (reading number 0), with readings increasing in depth to approximate 4 cm (reading number 5,000). Measurements were undertaken in triplicate

**FIGURE A1:**
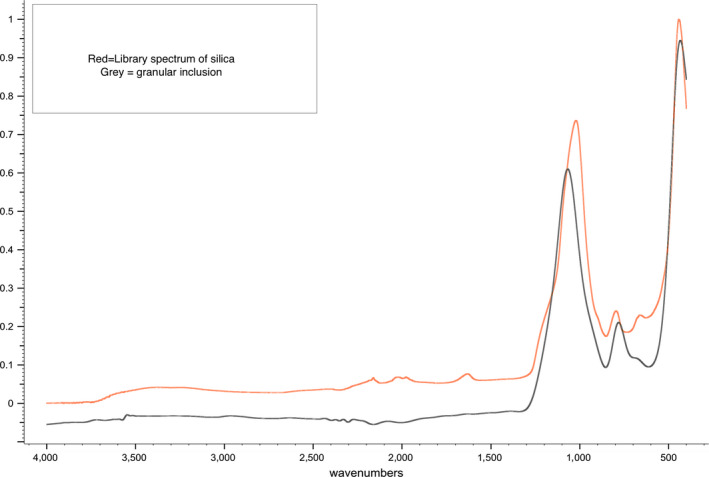
FTIR profile of *Pisolithus arhizus* inclusions versus silica standard, indicating that the inclusions rather than being pure sulfur as previously assumed are sulfur‐coated pure silica granules

Analysis of the soil pH from the Norris Annex collection plots (YNP) ranged from pH 1–3, while pH measurements of the interior of the *P. arhizus* sporocarps ranged from pH 4–5. GC‐MS analyses of the sporocarp biomass show a metabolome with a diverse array of alcohols, aromatic hydrocarbons, fatty acid methyl esters, saturated hydrocarbons, and long and short‐chain fatty acids (Table A1). ICP‐MS analysis of the *P. arhizus* sporocarp and parent soils indicate that *P. arhizus* uptakes a wide range of elements, and significantly concentrates Cu, Mg, Mn, Ni, and Zn relative to soils (Table 1). FTIR analysis shows the visible sporocarp inclusions are comprised of nearly pure silica, coated in elemental sulfur (Figure A1). Oxygen concentrations as a function of depth in the sporocarp were determined via oxygen microsensor. Over replicate measures we found oxic zones (up to 4× atmospheric concentrations) directly adjacent to zones of anoxia (Figure 2). There were no consistent patterns of high O_2_ versus low O_2_, (e.g., peaks were not at specific depths) indicating that results were not simply an artifact of the delivery of the probe through the *P. arhizus* individuals.

**TABLE 1:** ICP‐MS analysis of *Pisolithus arhizus* sporocarp (*n* = 9) and their resident soils (*n* = 9)

	Al	As	Ba	Cd	Cr	Co	Cu^†^	Fe	Pb
Soil	627 (205)	18 (6)	31 (10)	0.08 (0.03)	1 (0.1)	0.5 (0.02)	0.8 (0.3)	582 (226)	5.4 (3)
*Piso*	48 (17)	0.78 (0.3)	1.3 (0.4)	0.14 (0.05)	0.7 (0.3)	0.04 (0.01)	14.2 (2)	84 (23)	0.4 (0.08)

	Li	Mg^†^	Mn^†^	Mo	Ni^†^	Se	Sr	Zn^†^	V
Soil	0.6 (0.2)	45 (21)	2 (0.6)	1.5 (0.6)	0.4 (0.1)	0.7 (0.2)	0.6 (0.2)	1 (0.4)	1 (0.4)
*Piso*	0.5 (0.2)	954 (100)	6 (1)	0.6 (0.2)	3 (0.1)	0.3 (0.2)	0.3 (0.05)	52 (7)	0.12 (0.04)

Note

Mean elemental concentration: mg/kg and (*SE*).

^†^ (*p* < .0001) denotes a significant enriched in the *P. arhizus* sporocarp (*T* test), ^††^ *p* < .05.

### 3.2 Molecular analyses

**FIGURE 3:**
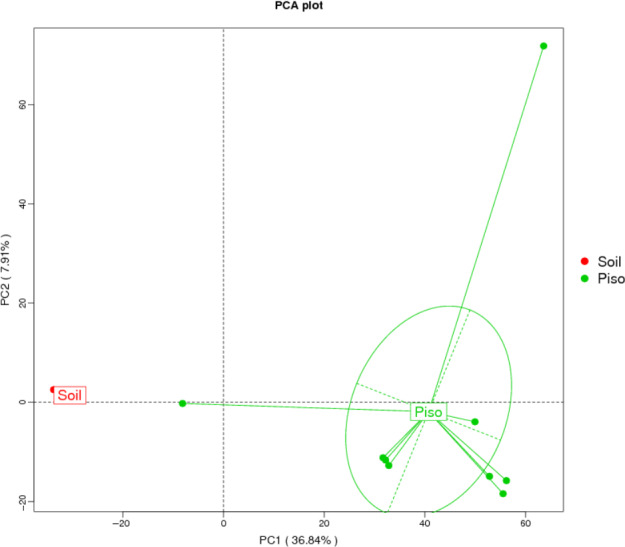
Principal component analysis (PCA) of the *Pisolithus arhizus* microbiome and parent soil communities. Analysis indicates that the microbiomes of each are comprised of distinct populations of microbes

**FIGURE 4:**
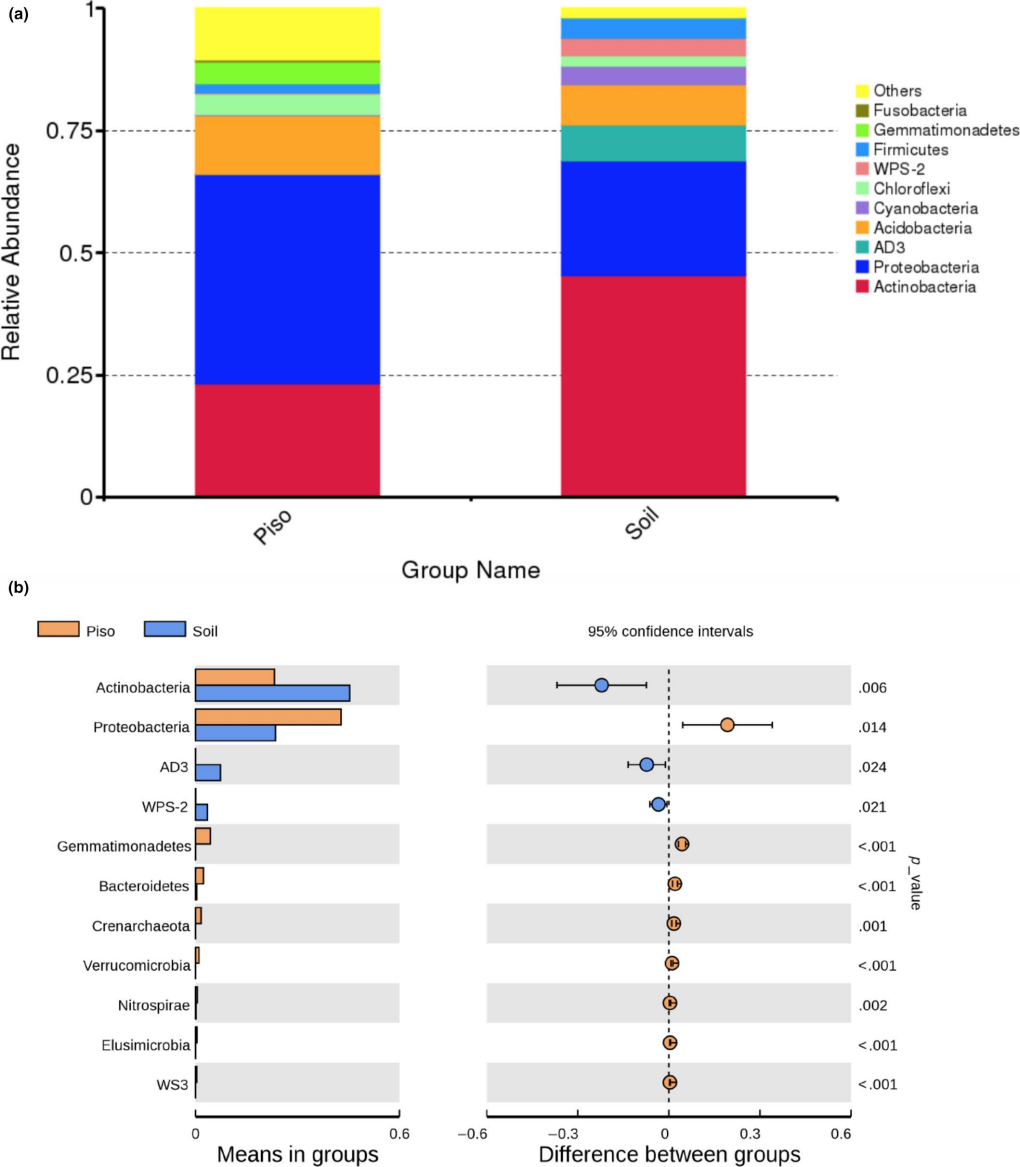
Phylum‐level abundances and statistical analysis comparison of the *Pisolithus arhizus* microbiomes to soil microbial communities. (a) comparative relative abundances of microbial phyla in soils and *P. arhizus*, (b) Mann–Whitney *U* test results of community differences at the phylum level

**FIGURE A2:**
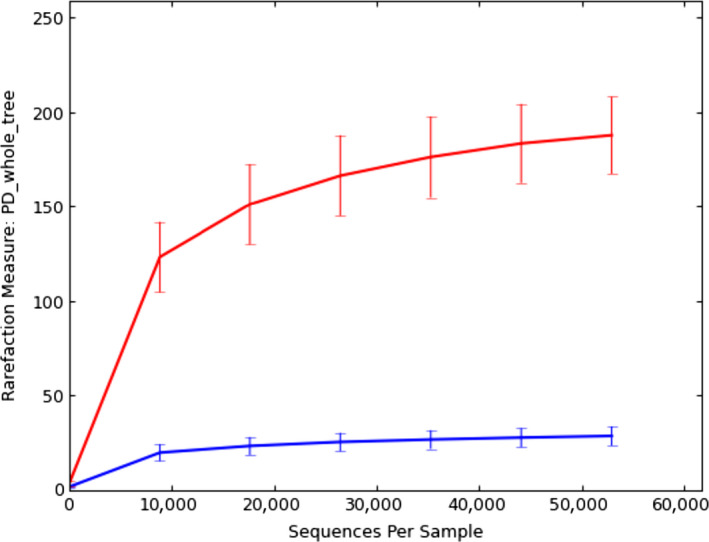
Rarefaction measure of species richness of the microbiome of *Pisolithus arhizus* (upper) versus that of the parent soil community (lower). This measure indicates greater species richness in the *P. arhizus* microbiome

**FIGURE A3:**
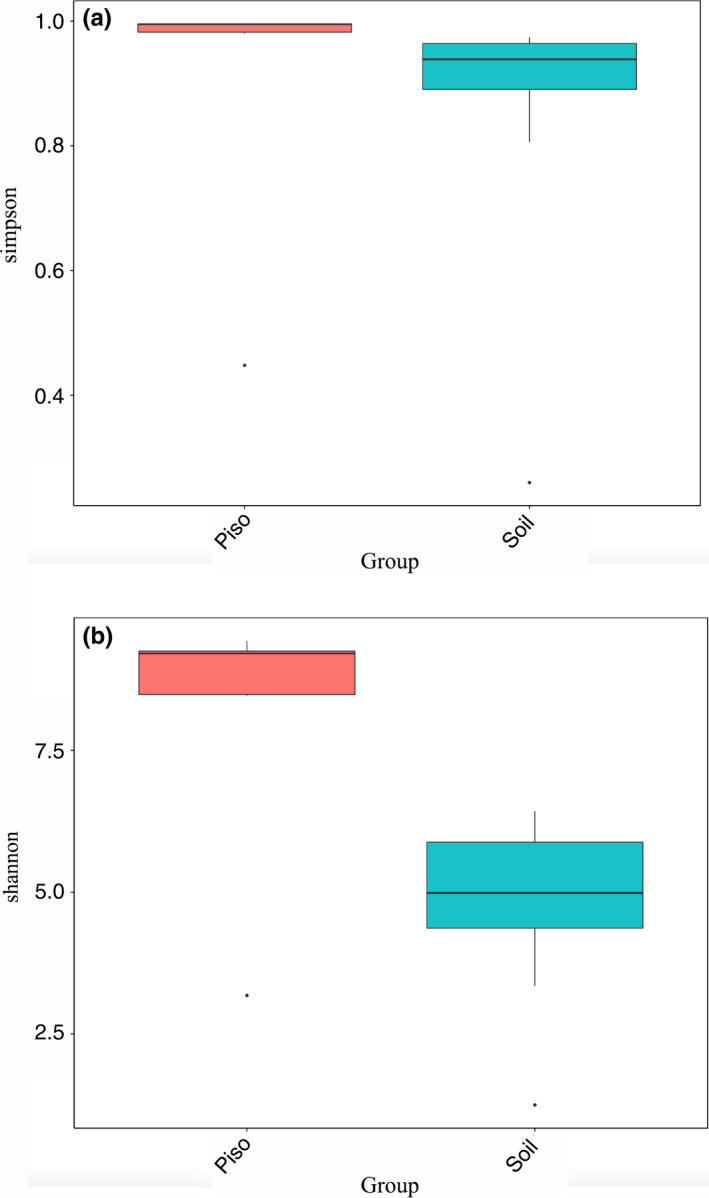
Diversity measures comparing *Pisolithus arhizus* communities to those in parent soils: Simpson's significant to *p* < .002 (a) and Shannon significant to *p* < .01 (b). All analyses indicate a community within *P. arhizus* individuals that are significantly different from and more diverse than that of parent soils

All ITS‐2 amplicons (387 bp) from the *P. arhizus* specimens sampled in this study had identical sequences (Appendix 2A), indicating that a single species (*P. arhizus*) was used in this study. These data have further supported the uniform observed sporocarp morphologies of the specimens (data not shown) and are consistent with our previous studies (Cullings & Makhija, 2001). Assessment of the 16S rRNA gene sequence diversity of the microbiological communities of both the *P. arhizus* sporocarps and parent soils show that the communities were comprehensively sampled with 90K–120K unique itags in all samples (Good's Coverage >99.5%). The diversity of the microbial communities of the *P. arhizus* microbiome and that of parent soils are significantly different in composition, with the *P. arhizus* microbiome being both higher in species richness and more diverse (Table 2, Figures 3, 4, A2, A3).

**TABLE 2:** Statistical tests of differences/similarities between *Pisolithus arhizus* and soil microbiomes

Test	Degrees of freedom	*F*/*R* value	*p*
Bray Adonis	1 (19)	13.329	<.001
AMOVA	1 (19)	16.1551	<.001
ANOSIM		0.99	<.001

Taxonomic comparisons demonstrate that microbiome and soil communities are dominated by Actinobacteria, Proteobacteria, Dormibacteraeota AD3), Acidobacteria, Cyanobacteria (as chloroplasts), Chloroflexi, Firmicutes, Gemmatimonadetes, and Fusobacteria (Figure 4a). However, Mann–Whitney *U* test analysis confirmed that the communities at the phylum level are significantly different (Figure 4b) with the differences being due to variation in the Actinobacteria and Dormibacteraeota (AD3) which were more abundant in soils, and Proteobacteria, Eremiobacteraeota (WPS2), Gemmatimonadetes, Bacteroidetes and Crenarchaeota being more abundant in the *P. arhizus* sporocarp. Also, while in relatively low relative abundance in the overall *P. arhizus* community, OTUs from the phyla Verrucomicrobia, Nitrospirae, Elusimicrobia, and Latescibacteria (WS3) were statistically more abundant than in soils. The proteobacterial and actinobacterial together comprise 66% and 68% of the two communities respectively.

The differences in the Proteobacteria populations in soils and *P. arhizus* sporocarp samples are largely due to the presence of *Burkholderia* in the *P. arhizus* microbiome. *Burkholderia* OTUs are virtually absent from soils (mean/*SE* in *P. arhizus* 8,943 (6,067), mean in the soil is 5 (4), *p* < .001). Conversely, the actinobacterial genus *Mycobacterium* dominates the soil samples (21% of the community) but is absent from the *P. arhizus* microbiomes altogether. Of note, among the top 10 phyla in the *P. arhizus* microbiome are cyanobacterial sequences that share a similarity, albeit distant, with the chloroplasts of the green alga in the genus, *Koliella* (Appendix 2B). These are present in all *P. arhizus* individuals but not detected in soils, and may explain the relatively high O_2_ zones present in *P. arhizus* individuals (Figure 2).

Sequences from our PCR‐cloning protocols were used to make rough functional approximations. These were done by comparisons of cloned sequences to sequences in GenBank (Table A2). Attributes were inferred from taxa with closest GenBank matches of 95% or greater sequence similarity and 98%–100% coverage.

Three of the most abundant microbes inhabiting the *P. arhizus* microbiome could not be classified to described bacterial or archaeal groups, or to previously described members of the CPR. These sequences comprised 2%–7% of the overall community. For further characterization of these signatures, we employed the cloning methods and controls described above to obtain a more detailed look at their origins. Pairwise comparisons of resultant sequences indicate that they may be distinct entities in their own right (Table A3). These comparisons indicated two groups, one comprised of two sequences (KU527064.1 and KU527065.1) that is most similar to the CPR candidate phylum CPR2 and a third OTU (KT599128.1) that shares nearly equal similarity with members of a monophyletic group in the Parcubacteria comprised of the Wolfebacteria, Jorgensenbacteria, and Giovannonibacteria (Hug et al., 2016). The percent similarity values among these four are within the average and minimum similarity ranges that define individual phyla using this gene (Yarza et al., 2010).

## 4 DISCUSSION

Microbe/fungal symbioses are relatively ancient, and recent fossil evidence indicates that fungi have contained microbial symbionts since their inception over a billion years ago (Bengtson et al., 2014, 2017; Mulkidjanian, Bychkov, Dibrova, Galperin, & Koonin, 2012). Investigations into the function and evolution of these symbioses have shown that bacterial symbionts of AM systems of the Glomeromycota can exhibit genome reduction and loss of genes coding for an array of energy pathways in response to carbon received from the phytobiont (e.g., Ghignone et al., 2012; Lackner, Moebius, Partida‐Martinez, Boland, & Hertweck, 2011; Naito et al., 2015). Studies of mushroom‐forming basidiomycete fungi indicate the fruiting bodies of these fungi can support diverse microbiomes as well with both mode of nutrition playing significant roles in controlling the composition of their microbiomes (Barros et al., 2007, 2008; Pent et al., 2018; Rinta‐Kanto et al., 2018). Our data demonstrate that the *P. arhizus* system is unique to those of previous studies in several ways. Not only is *P. arhizus* a basidiomycete with a closed fruiting body which houses steep oxygen transitions, but the fruiting bodies also contain two additional energy sources not considered in systems studied previously. Namely, the rich hydrocarbon slurry that is present in the *P. arhizus* environment, and the enrichment in several elements indicated by ICP‐MS analysis. This unique combination of conditions and energy sources could provide insight into factors that influence microbiome community structure and dynamics and also into strategies both genomic and functional that a community of symbionts could employ to adjust to different selection pressures.

Our molecular analyses demonstrate that the *P. arhizus* microbiome is comprised of a rich and diverse microbial community. The apparent relative dominance of the betaproteobacterium, *Burkholderia,* is interesting, and its presence could provide a range of functional advantages. Burkholderia spp. are known to form stable and ancient endosymbioses with insects, plants, and fungi (Compant, Nowak, Coenye, Clement, & Ait Barka, 2008; Nguyen & Bruns, 2015). *Burkholderia* phylotypes have been detected in acidic YNP hot springs at temperatures to 89°C where they appear to be involved in nitrogen fixation and aromatic hydrocarbon degradation (Hamilton, Lange, Boyd, & Peters, 2011). Further, when in symbiosis with plants *Burkholderia* species can enhance the ability to breakdown aromatic hydrocarbons (a trait that would be useful in the *P. arhizus* environment), can aid growth and nodulation, and can also impact the genome of associated plants via horizontal gene transfer (Bontemps et al., 2010; Nguyen & Bruns, 2015; Suarez‐Moreno et al., 2012). It has been hypothesized that the common ancestor of symbiotic *Burkholderia* was a diazotroph and *Burkholderia* is known to be a functionally important nitrogen‐fixing component of ectomycorrhizal associations with conifers (Andreolli et al., 2013; Bontemps et al., 2010). Further, *Burkholderia phymatum* is a highly effective nitrogen‐fixing symbiont of *Mimosa* spp. that fixes nitrogen *ex planta*. This broad diazotrophic trait could provide a mechanism for a microbially based mode of nitrogen acquisition by *P. arhizus*. This would be particularly relevant in this case as we have shown previously that the soils in which *P. arhizus* grows are extremely nutrient‐poor (Cullings & Makhija, 2001). We had hypothesized in this previous study that *P. arhizus* could obtain nitrogen via the saprophytic activity of woody debris in these soils. However, nitrogen acquisition via symbiosis with an efficient diazotroph such as *Burkholderia* provides an equally plausible mechanism.

Interestingly, our PCR‐cloning methods revealed the presence of a sequence most similar (93.7% similarity, 99% coverage) to the chloroplasts of the green alga *Koliella* in the *P. arhizus* microbiome (Appendix 2B). Though the similarity to *Koliella* is relatively low, the presence of a green alga would provide a plausible explanation for the presence of elevated O_2_ in regions of the *P. arhizus* fruiting body. *Koliella* is a marine alga that is adapted to very low light conditions. It is capable of rapid response to light fluctuations to maintain function in extreme low light conditions, an adaptation that undoubtedly enhances survival during periods of prolonged Arctic and Antarctic darkness (Ferroni et al., 2007; La Rocca et al., 2015). Studies of the response of *Koliella* to heavy metal contamination (e.g., cadmium at concentrations as low as 1 ppm) indicate that they can show rapid and profound degradation of structure and function in response to the presence of the metals (La Rocca, Andreoli, Giacometti, Rascio, & Moro, 2009). The observation that the alga present within *P. arhizus* can live and metabolize in an environment that is rich in toxic metals and aromatic hydrocarbons suggests unique physiology as compared to the algal taxon currently most similar in GenBank.

The absence of *Mycobacterium* spp. (the dominant *Actinobacteria* strain in parent soils) in the *P. arhizus* microbiome is perhaps surprising. *Mycobacterium* strains have been detected in YNP hot springs and soils at Norris Geyser Basin (the location of our study) where they can grow at pH 1–3 and elevated temperatures (Santos, Fernandes, Fernandes, Oliveira, & Cadete, 2007; Walker, Spear, & Pace, 2005). They are widespread in soils and water where they can be important disease‐causing agents (Primm, Lucero, & Falkinham, 2004). Some mycobacterial strains can degrade polycyclic aromatic hydrocarbons (PAH) (Miller et al., 2004; Uyttebroek et al., 2006), therefore their physiology coupled with conditions within the *P. arhizus* fruiting body would seem to support their growth therein. However, just the opposite appears true; *P. arhizus* sporocarp microbiomes contained no detected mycobacterial signatures. What mechanism is at work to inhibit their growth whether passive (e.g., metal toxicity) or active (e.g., an exclusion mechanism on the part of *P. arhizus*) is unclear. *Mycobacterium* tend to be highly resistant to antibiotics, making infections by this bacterium difficult to eliminate (Nguyen & Thompson, 2006). It has been hypothesized that the antibiotic resistance mechanisms employed by *Mycobacterium* could be inhibited pharmaceutically (Danilchanka, Pavlenok, & Niederweis, 2008) and *P. arhizus* may be employing a similar antibiotic resistance‐inhibiting trait to exclude mycobacterial strains from its sporocarp. Further metabolomics study could help locate such a compound, should it exist.

In addition to the abundant Betaproteobacteria, there were several phyla that though present at relatively low frequency their pattern of distribution was consistent enough that they were significantly more abundant in *P. arhizus* than in parent soils. These include Gemmatimonadetes, Nitrospirae, Bacteroidetes, Elusimicrobia, Latescibacteria (WS3), and Verrucomicrobia. The Gemmatimonadetes are a diverse group found in soils and near‐shore sandy sediments (Conte et al., 2018; Huang et al., 2019). These have recently been found to be a component of deep anoxic seafloor sediments (Fang et al., 2019) and contain the genetic pathways to fully reduce sulfate (Baker, Lazar, Teske, & Dick, 2015). The Nitrospirae is a group of nitrifying bacteria that is comprised at least in part by sulfate‐reducing thermophiles (Baker et al., 2015; Bhatnagar et al., 2015) and magnetotactic bacteria (Islam, Peng, & Ali, 2018; Lefevre, Frankel, Abreu, Lins, & Bazylinski, 2011; Lin, Li, & Pan, 2012). Nitrospirae have also been implicated in woody biomass conversion, a process that requires the breakdown of complex phenolic‐based hydrocarbons (Vishnivetskaya et al., 2015). Also included as microbiome dominants are the Bacteroidetes, another widespread group that is prevalent as mutualists in gut microbiomes. Members of this group are also found in soils, ocean, and freshwater where they are important recyclers of complex biopolymers via extracellular enzyme activity (Thomas, Hehemann, Rebuffet, Czjzek, & Michel, 2011). The Elusimicrobia comprise a deeply diverging group within Bacteria and are common in the hindgut of termites where they are presumably active in the degradation of a woody substrate (Brune & Ohkuma, 2010). Although some strains are obligate anaerobes others can be free‐living and inhabit a diverse array of habitats including soils and sediments, contaminated aquifers (Geissinger, Herlemann, Morschel, Maier, & Brune, 2009; Herlemann, Geissinger, & Brune, 2007; Zheng & Brune, 2015). The Latescibacteria (WS3) was first discovered in anoxic/hydrocarbon‐rich lake sediments (Youssef et al., 2015). These have since been found in varied settings including marine, freshwater, terrestrial, bioremediation, and symbiotic settings (Markowitz et al., 2014). Their functionality includes genes coding for dockerins within cellulosomes and members of this group have been detected in metagenomes of anoxygenic microbial mats in YNP where these gene assemblies would enable the breakdown of complex hydrocarbons (Farag, Youssef, & Elshahed, 2017). And, finally, the phylum Verrucomicrobia, represented here primarily by members of the Chthoniobacterales and the Verrucomicrobiales. The former has recently been detected in lichens (Cernava et al., 2017). Some members of this group can survive and protect their hosts in conditions of high oxidative stress and play a role in carbon cycling via aromatic hydrocarbon degradation. They may also provide antibiotic resistance. Any or all of these potential functions would be advantageous in this environment.

In addition to these known phyla, our data included OTUs that could not be classified into phylum. Pairwise % similarity comparisons indicate that these comprise sister lineages to known groups in the CPR. The CPR comprises more than 15% of all microbial life and contains greater than 70 newly discovered phyla (Brown et al., 2015). Organisms in this broad radiation are known only from culture‐independent genetic surveys, which can have greatly reduced genomes that lack many biosynthetic pathways required for independent growth and some have correspondingly ultra‐small cells (Danczak et al., 2017; Paul et al., 2017). Some have 16S rRNA genes that are sufficiently divergent to avoid detection using PCR‐based methods and are therefore known only through analyses of genomic reconstructions (Brown et al., 2015). The physiology and life history strategies of these organisms are only now coming to light. However, recent genomics studies indicate that most have reduced biosynthetic potential and the genomes of many contain diversity‐generating retroelements which are thought to provide a level of physiological flexibility that would favor their success as endosymbionts (Brown et al., 2015; Paul et al., 2017).

Although the percent similarity between our lineages and known members of the CPR fall within the ranges that define known phyla (Yarza et al., 2010), whether these sequences represent new phyla in their own right could be the subject of debate. Recent efforts to resolve issues such as paraphyly of traditional phyla have introduced a microbial taxonomy classification based on phylogenetic depth. This strategy conflates the CPR into a single, large phylum (the Patescibacteria) thus relegating all phyla within to the class level (Parks et al., 2018). Further, given that our findings are based on only roughly half to two‐thirds of the complete 16S rRNA gene, caution must be employed when assigning classification. Considerable power could be conferred to these preliminary indications by using full genome reconstruction from metagenomics analysis. This method has become more common in recent years and has been instrumental in restructuring the Tree of Life both through rigorous analysis of known genomes from environmental samples and also through the discovery of groups (e.g., the highly divergent Wirthbacteria) that are sufficiently divergent to lack a detectible 16S rRNA gene (Brown et al., 2015; Hug et al., 2016). Whether ultimately classified as novel classes or phyla, it is clear that our microbes represent novel groups at what is traditionally considered to be at the phylum level within the CPR. We plan to utilize metagenomics methods to further investigate the evolution of these novel microbes inhabiting the *P. arhizus* microbiome.

These results demonstrate that the fruiting bodies *of P*. arhizus create and provide a habitat that conducive to microbial growth, essentially an “island” in an otherwise hostile environment. Further, the fruiting bodies house a rich and diverse microbiome that is comprised not only of known bacterial lineages but also novel microbes. What roles these microbes play in the *P. arhizus* environment is not yet known. They may provide services similar to those observed in the few bacterial/mushroom systems studied previously, for example, aiding in fruiting body formation, and enhancing mycelial growth and nitrogen acquisition. Though the *P. arhizus* strain inhabiting Yellowstone geothermal soils is not ectomycorrhizal (Cullings & Makhija, 2001) most other strains are, including those inhabiting thermal areas in New Zealand (Moyersoen & Beever, 2004). Laboratory experiments pairing specific strains of pseudomonad “helper” bacteria to *P. albus* suggest that these bacteria, which are associated with ectomycorrhizal roots, can aid in mycorrhizal formation (Founoune et al., 2002). We are conducting similar synthesis experiments targeting the ectomycorrhizal *Pisolithus* strains and their host trees in New Zealand to determine if similar processes are at work that system. It is also possible that additional benefits arise from metabolisms that can take advantage of the unique set of conditions inherent to the *P. arhizus* environment, for example, the sharp oxygen transitions which exist close to complex elemental combinations. Benefits could also come via energy production through chemosynthetic processes involving the array of elements detected within *P. arhizus*. Metabolism of sulfur is one obvious candidate process. Early studies of *P. arhizus* physiology indicated that this fungus is capable of enzymatic conversion of elemental sulfur to sulfate (Grayston & Wainwright, 1988). However, the presence of microorganisms in the *P. arhizus* microbiome apparently capable of performing this function suggests that this activity might be due to microbial activity as much as to any innate ability on the part of *P. arhizus*. Energy from the microbiome could also be obtained directly from the enzymatic breakdown of the diverse hydrocarbons measured within the *P. arhizus* fruiting body, or indirectly by providing carbon substrate for the polymer‐degrading enzymes that are inherent to basidiomycete fungi (Reviewed by Lundell, Mäkelä, & Hildén, 2010 **)**. The potential for any or all of these processes appears to be present in the *P. arhizus* microbiome. We realize, however, that the similarity‐based method we employed to estimate functional diversity provides only rough estimates of functionality. Therefore, to more fully elucidate functional diversity and evolutionary history of the microorganisms residing within the *P. arhizus* microbiome, we are planning combined metagenomics/metatranscriptomic experiments to obtain annotated genomes for functional potential and phylogenetic analyses.

## CONFLICTS OF INTEREST

None declared.

## AUTHOR CONTRIBUTION

**Ken Cullings:** Conceptualization (lead); Data curation (lead); Formal analysis (lead); Funding acquisition (lead); Investigation (lead); Methodology (lead); Resources (lead); Software (equal); Supervision (lead); Validation (lead); Visualization (lead); Writing‐original draft (lead). **Matthew B. Stott:** Conceptualization (supporting); Formal analysis (supporting); Investigation (supporting); Methodology (supporting); Writing‐original draft (supporting); Writing‐review & editing (supporting). **Nicole Marinkovich:** Data curation (equal); Formal analysis (equal); Investigation (supporting); Methodology (equal); Writing‐review & editing (supporting). **Julia DeSimone:** Data curation (equal); Formal analysis (equal); Investigation (equal); Methodology (equal); Writing‐review & editing (supporting). **Shilpa Bhardwaj:** Formal analysis (equal); Methodology (equal).

## ETHICS STATEMENT

None required.

## Data Availability

This Targeted Locus Study project has been deposited at DDBJ/ENAL/GenBank under the accession KDWD00000000. The version described in this paper is the first version, KDWD01000000. Data obtained by PCR/cloning/sequencing have been made available through GenBank, with accessions KT599104–KT599137 and KU527064–KU527065.

## APPENDIX 1

**TABLE A1:** Nonquantitative assessment of major chemical groups^a^ within *Pisolithus arhizus* individuals

Major chemical class	Compound
Alcohols	Alcohols: 1‐octen‐3‐ol; 2‐octen‐1‐ol, (Z)‐; 1‐octanol; 1‐heptanol; phenylethyl alcohol, 3‐octanol; Aldehydes: 2‐undecenal; 2‐thiophenecarboxaldehyde; nonanal; trans‐4,5‐Epoxy‐(E)‐2‐decenal; 3‐thiophenecarboxaldehyde; benzaldehyde, 2,4‐dimethyl‐, benzaldehyde; 2‐methoxy‐, 2,4‐nonadienal; octanal; 2,4‐decadienal; (E,E)‐butanal; 3‐methyl‐, 2‐octenal; (E)‐2‐nonenal; (E)‐hexanal; benzaldehyde; 4‐hydroxy‐, 5‐ethylcyclopent‐1‐enecarboxaldehyde; 5‐methyl‐2‐thiophenecarboxaldehyde; propanal; 2‐methyl‐, 2‐phenylpropenal
Aromatic hydrocarbons	Benzofuran; 2,3‐dihydro‐, trans‐1,10‐dimethyl‐trans‐9‐decalol; 2‐n‐octylfuran; 2furanmethanol; acetophenone; m‐guaiacol; benzene; 1‐ethyl‐1‐propenyl‐, furan; 2‐hexyl‐, furan; 2‐pentyl‐, benzonitrile; acetophenone; 4'‐hydroxy‐, vinyl trans‐cinnamate; 2‐propenoic acid; 3‐phenyl‐, methyl ester, (E)‐ muscimol; benzeneacetic acid
Fatty acid methyl esters	9,12‐Octadecadienoic acid (Z,Z)‐, methyl ester; 9‐octadecenoic acid (Z)‐, methyl ester; hexadecanoic acid, methyl ester; methyl hexadec‐9‐enoate; pentadecanoic acid, methyl ester; methyl tetradecanoate; nonanoic acid, methyl ester
Branched/unsaturated hydrocarbons	(3‐Nonen‐5‐yne, 4‐ethyl‐)
Saturated hydrocarbons	1,5‐Hexadien‐3‐yne Ketones: 2‐undecanone; 3‐octanone; 2,4‐octadienal, (E,E)‐
Long‐chain fatty acids	n‐Hexadecanoic acid; pentadecanoic acid Organosulfur compounds: 1,3‐butadiene; 1‐(ethylthio)‐, methional; dimethyl trisulfide; dimethyl disulfide
Short‐chain fatty acids	Nonanoic acid; 2‐octenoic acid; octanoic acid, 2‐octenoic acid, (E)‐; hexanoic acid, acetic acid.

^a^ For the sake of brevity his list is not exhaustive but is restricted to those compounds that could be definitively identified through the database, and illustrates the fact that the metabolome is comprised of a rich assemblage of organic compounds.

**TABLE A2:** Functional approximations of OTUs^a^ detected in *Pisolithus arhizus* sporocarp microbiomes

Taxon	Characteristics of closely related strains
Actinobacteria	Filamentous, mesophilic, and acidophilic facultative anaerobes; sulfur, iron, and pyrite oxidizers; iron‐reducing thermoacidophiles
Proteobacteria	Bacteriochlorophyll‐containing acidophiles; aliphatic hydrocarbon metabolizers; anaerobic chemolithotrophic sulfur oxidizers; and thermophilic sulfate reducers
Chloroflexi	Thermophilic, anaerobic halophiles
Acidobacteria	Anoxygenic photoheterotrophs; chemoorganotrophic acidophiles

Note

Functional approximations were undertaken where *Pisolithus arhizus* community OTU similarities where >95% (98%–100% coverage) with the16S rRNA gene sequences with characterized microbial strains.

^a^ Accession numbers for OTUs used for this approximation: KT599104–KT599137.

**TABLE A3:** Clonal 16S rRNA gene sequence similarities^a^ between unknown *Pisolithus arhizus* sporocarp microbiome representatives and representatives from the CPR superphylum

Accession/Phylum	Closest CPR superphylum and percent similarity		
KU527064.1 and KU527065.1	CPR−2:82%		
	Giovannonibacteria	Wolfebacteria	Jorgensenbacteria
KT599128.1	84%	83%	83%
Giovannonibacteria	–	83%	85%
Wolfebacteria	–	–	85%

Abbreviation: CPR, candidate phylum radiation.

^a^ Percent similarities (with 850–900 bp, 95%–99% query cover) defining phylum‐level relationships using 16S rRNA gene comparisons (Yarza et al., 2010): Maximum: 84.7% ± 1.9; Average: 81.7% ± 1.8; Minimum: 78.4% ± 2.0

## APPENDIX 2

**A**: Consensus ITS‐2 reference sequence obtained from *Pisolithus arhizus* fruiting bodies. All ITS‐2 sequences obtained in this study were identical.

GAATCGCGATAAGTAATGTGAATTGCAGATTTTCCGTGAATCATCGAATCTTTGAACGCACCTTGCGCTCCTTGGTATTCCGAGGAGCATGCCTGTTTGAGTGTCATCGAAATCTCAAACCAAGCTTTTCTTGACTTCGGTCGAAGGCTCGGGTTTGGACCGTTGGGAGTCTGCGGGCGACGCATGTCGTCGGCTCTCCTGAAATGCATTAGCGGTGGGCATGCAAGTCTTGCTTGGCACCAGCCTCTCCCGGCGTCATAGTGATCGTCGCGGGCTGTCCAGCTGCAAGGGACATGTCCCATGCTTCTCCAACTTTGCGAGCCCTCTCCTGGGCTCTGCGTTCGAAGGCTTGACCTCAAATCAGGTAGGACTACCCGCTGAACTTAA

**B**: Partial 16S rRNA gene sequence obtained by PCR/cloning/sequencing overlapped and checked, and includes hypervariable regions V6‐V9. The sequence has the highest sequence similarity with the chloroplast 16S rRNA gene sequences in the algal genus *Koliella*.

CAAAGGTTAAAGTTACGACTTTGGGCCAAGCCGATTCTGGTAGTGTGACGGGCGGTGTGTACAAGGCCCGGGAACGTATTCACCGCTGTATGGCTGACCAGCGATTACTAGCGATTCCGACTTCATGCAGGCGAGTTGCAGCCTGCAATCCGAACTGAGACCGGGTTTTTGAGGTTAGCTTGCTCTCGCGAGGTTGCATCTCATTGTCCCGGCCATTGTAGCACGTGTGTCGCCCAGGGTATAAGGGGCATGCTGACTTGACGTCATCCTCACCTTCCTCCGGCTTATCACCGGCAGTCTCTCTAGTTTCCCACAAATATGTATATTTCGGCAACTAAAGACAAGGGTTGCGCTCGTTGCGGGACTTAACCCAACATCTCACGACACGAGCTGACGACAGCCATGCACCACCTGTGTCCACCTTCTTCTGCACGAATGTGTAGAAGATACGCTCTGACTTTCATCTGAGTTAGTGGGCATGTCAATCCCCTGGTAAGGGTTCTTCGCGTTGCANCGAATTAAACCACATGCTCCACCGCTTGTGCGGGCCCNCGTCAATT
